# Improper Tagging of the Non-Essential Small Capsid Protein VP26 Impairs Nuclear Capsid Egress of Herpes Simplex Virus

**DOI:** 10.1371/journal.pone.0044177

**Published:** 2012-08-31

**Authors:** Claus-Henning Nagel, Katinka Döhner, Anne Binz, Rudolf Bauerfeind, Beate Sodeik

**Affiliations:** 1 Institute of Virology, Hanover Medical School, Hanover, Germany; 2 Institute of Cell Biology, Hanover Medical School, Hanover, Germany; Queen’s University, Canada

## Abstract

To analyze the subcellular trafficking of herpesvirus capsids, the small capsid protein has been labeled with different fluorescent proteins. Here, we analyzed the infectivity of several HSV1(17^+^) strains in which the N-terminal region of the non-essential small capsid protein VP26 had been tagged at different positions. While some variants replicated with similar kinetics as their parental wild type strain, others were not infectious at all. Improper tagging resulted in the aggregation of VP26 in the nucleus, prevented efficient nuclear egress of viral capsids, and thus virion formation. Correlative fluorescence and electron microscopy showed that these aggregates had sequestered several other viral proteins, but often did not contain viral capsids. The propensity for aggregate formation was influenced by the type of the fluorescent protein domain, the position of the inserted tag, the cell type, and the progression of infection. Among the tags that we have tested, mRFPVP26 had the lowest tendency to induce nuclear aggregates, and showed the least reduction in replication when compared to wild type. Our data suggest that *bona fide* monomeric fluorescent protein tags have less impact on proper assembly of HSV1 capsids and nuclear capsid egress than tags that tend to dimerize. Small chemical compounds capable of inducing aggregate formation of VP26 may lead to new antiviral drugs against HSV infections.

## Introduction

**Figure 1:**
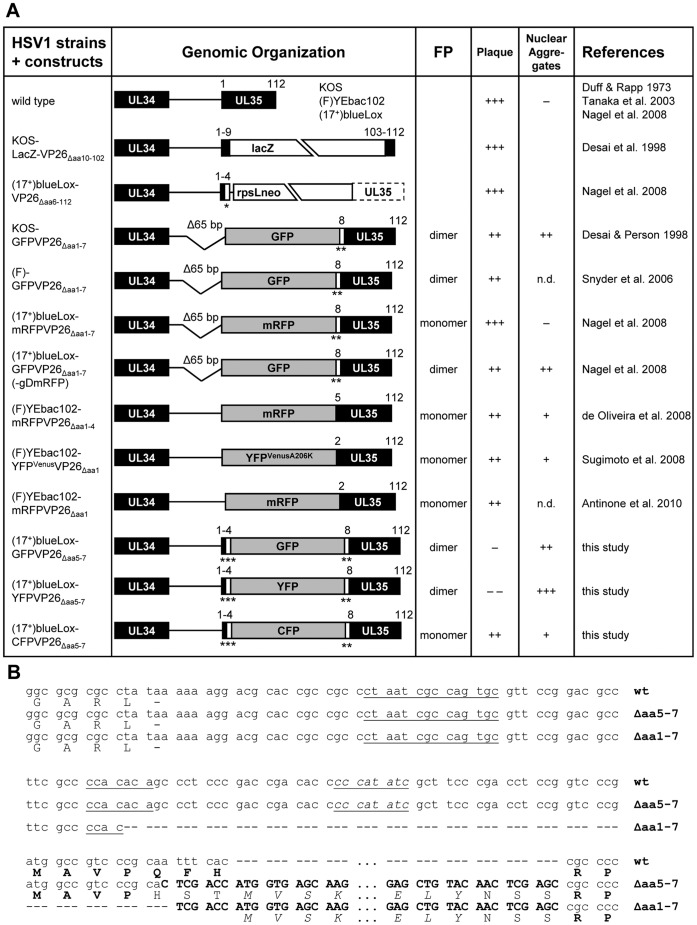
HSV1-VP26 constructs. (A) 1^st^ column: HSV1 constructs in which the SCP VP26 has been tagged with different fluorescent protein domains. 2^nd^ column: Genomic organization of the UL35 region approximately drawn to scale. The gene UL35 coding for VP26 has been disrupted by replacing it with lacZ or an rpsLneo cassette out of frame. Some constructs lack a 65 bp region upstream of UL35 (D65 bp) including the first seven N-terminal codons of VP26 (Daa1–7), while others lack only four (Δaa1–4) or just one (Daa1) codon. For the present study, the fluorescent protein tag was inserted between VP26 residues 4 and 8 (Daa5–7). Due to the mutagenesis, some strains contain additional linkers (*, AW; **, NSS; ***, HST). 3^rd^ column: Propensity of the fluorescent protein (FP) to dimerize [76]–[80]. 4^th^ column: Ability of the construct to replicate and to form plaques (+++, similar to wild type; ++ attenuated, but robust growth; − strongly attenuated, tiny plaques; −−, single fluorescent cells, no plaques). 5^th^ column: Propensity of the construct to induce nuclear aggregates (+++, large irregular shaped aggregates; ++, large aggregates early after infection or transfection; +, aggregates late in infection; − aggregates in less than 2% of cells even late in infection). 6^th^ column: References. (B) Nucleotide (upper lines) and amino acid (lower lines) sequences of the UL34/UL35 (pUL34/VP26) intergenic region. The 3¢ end of the UL34 ORF until the 5¢ start of the UL35 ORF are shown for wild type HSV-1, the GFPVP26_Äaa**5**–7_ (Äaa5–7) and GFPVP26_Äaa**1**–7_ (Äaa1–7) mutants. Additional nucleotides inserted during mutagenesis are shown in bold capitals, and the GFP amino acids are shown in italics. Putative Inr late promoter elements are underlined with the element perfectly matching the consensus sequence being underlined and in italics. The original amino acids encoded by UL35 are shown in bold capitals, the inserted GFP residues in italic capitals and the additional linker residues in normal script capitals.

Single and dual-color fluorescently tagged strains are valuable tools to elucidate the intracellular trafficking of virions and subviral particles. In an ideal case, the modified strain replicates with the same kinetics and to the same titers as its parental strain, and the tag neither interferes with any step of the viral life cycle, nor changes the biochemical properties of the modified viral structure. For herpesviruses, fluorescent protein (FP) domains attached to the small capsid protein (SCP) have been used extensively to characterize the molecular mechanisms of virus assembly and egress or nuclear targeting of incoming capsids in cells and biochemical assays (c.f. Fig.1; [1]–[13]). The SCPs are recruited to capsids via the major capsid proteins (MCP). Although similar building principles apply, the amino acid sequences of both, SCPs and MCPs vary considerably among the herpesviruses [14]–[18]. SCPs are essential for the replication of human and mouse cytomegalovirus, Epstein-Barr virus and Kaposìs sarcoma-associated herpesvirus, but not for the alphaherpesviruses herpes simplex virus type 1 (HSV1), pseudorabiesvirus (PrV) or varizella zoster virus (VZV; [7], [8], [19]–[23]). However, HSV1 strains lacking the SCP yield lower titers than wild type in the murine eye and trigeminal ganglion after corneal infection as well as in BHK cells [8], [20], [24]. PrV lacking the SCP is also less neuroinvasive and grows to lower titers in cell culture, while the SCP of VZV is essential for infection of the human skin xenograft murine model and of melanoma cells but not of embryonic lung fibroblasts [7], [25].

VP26, the SCP of HSV1, is a basic 12 kDa protein of 112 amino acid residues (aa) with low solubility and encoded by the gene UL35 [26], [27]. In solution, it is only 13 to 15% α-helical but is 80% β-sheet, and a secondary-structure algorithm predicts two α-helical regions between aa 13 to 31 and 42 to 72 [26], [28], [29]. Herpesvirus capsids are assembled in the nucleus and for its nuclear import VP26 requires the interaction with VP5, the MCP of HSV1, and either capsid protein preVP22a or VP19c [30]–[32]. Hexamers of VP5 form the 150 hexons on the faces and edges, while pentamers of VP5 form the 11 pentons on the vertices of the icosahedral capsid. A virion can harbor up to 900 copies of VP26 as it decorates the top of the hexons in a hexamer [14], [16], [28]. The C-terminal half of HSV1-VP26, aa 50 to 112 are sufficient for binding to an interface of hydrophobic residues and small charged patches on the upper hexon domain [26], [33]. Combined cryoelectron microscopy and *ab initio* modeling suggest a novel fold of the C-terminal aa 42 to 112 with three short α-helices [29], [34]. While the hexons recruit VP26, the pentons serve as attachment sites for the tegument protein pUL36, and it has been suggested that this may be due to similarities between aa 66 to 96 of VP26 and aa 1712 to 1751 of pUL36 [14], [29], [35], [36].

In addition to VP5, HSV1-VP26 can also interact with the capsid proteins VP23 and pUL25 as well as the tegument proteins pUL11, pUL14, pUL16, pUL21, pUL37, VP16, pUL51, and pUS3 in yeast-two-hybrid assays [33], [37]. Yet, the incorporation of pUL37 and VP16 into HSV1 virions does not depend on VP26 but on pUL36 [38]. Furthermore HSV1-VP26 can bind to the host proteins tetraspanin-7 and the dynein light chains Tctex-1 and RP3 [33], [39], [40]. However, incoming capsids of HSV1-ΔVP26 can still utilize the microtubule motor dynein for transport to the nucleus, and HSV1-ΔVP26 capsids with inner tegument proteins on their surface can recruit dynein, whereas nuclear capsids devoid of inner tegument, but exposing VP26 cannot [5], [13], [20], [21], [41]. Thus, VP26 is not essential for binding dynein to viral capsids, but it may somehow regulate the interaction of dynein light chains with other host factors or the function of dynein.

**Figure 2:**
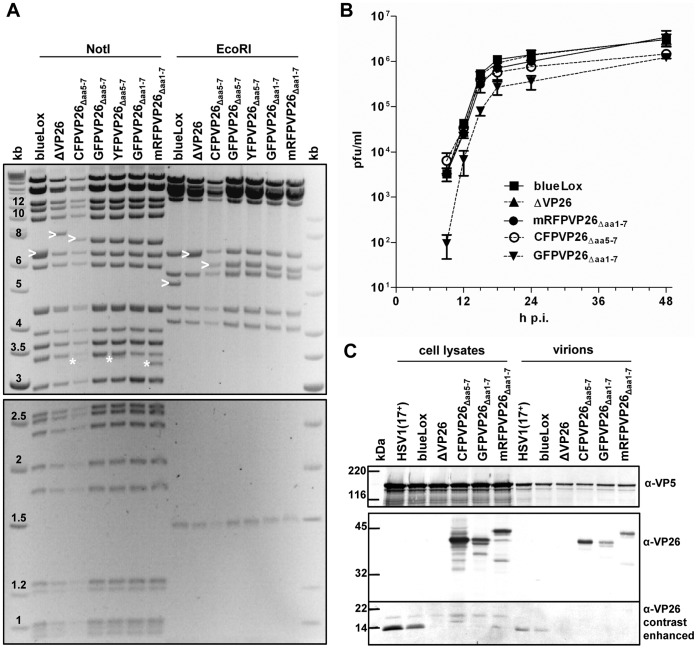
Characterization of HSV1(17^+^)blueLox-VP26 strains. (A) Restriction digestion analysis of different BAC clones with *Not*I and *Eco*RI. Band shifts resulting from modifications in the UL35 ORF (arrowheads) and the *Not*I joint fragments containing the viral a-sequences (asterisks) are indicated. DNA sizes in kb. (B) For single-step growth kinetics, Vero cells were infected in duplicates with 10 PFU/cell, and the amount of secreted infectious virus at a given time point was determined by duplicate plaque assays. (C) Vero cells were infected at an MOI of 10 PFU/cell with HSV1(17^+^) or HSV1(17^+^)blueLox and its derivatives as indicated. After 48 h, the cells and virions secreted into the culture medium were harvested, and analyzed by SDS-PAGE and immunoblotting for expression of VP5 (α-NC-1) and VP26 (α-VP26). The signals in the molecular weight range below 25 kDa were further contrast enhanced.

The N-terminal region of the VP26 of HSV1 or PrV has been successfully used to insert a FP while maintaining infectivity (c.f. Fig. 1A; [2], [4], [7], [8], [10], [11], [42], [43]). Cryoelectron microscopy tomography studies indicate that there are few highly ordered contacts between VP26 and the surrounding tegument, and most reported interaction partners employ its C-terminal half [24], [26], [33], [35], [36]. However the mass of the SCPs is tripled with additions such as mRFP (monomeric red FP) or GFP (green FP), CFP (cyan FP) or YFP (yellow FP) that have molecular weights of 25 to 27 kDa, and such tags can attenuate the replication of HSV1 and PrV in culture, and its pathogenesis in murine infection models [7], [8], [42], [44].

Since the HSV1(17^+^) strains that we had constructed previously exhibited prominent nuclear aggregates of GFPVP26 and reduced titers in BHK cells [8], [42], we analyzed here several other strains in order to identify a less invasive FP tag. While some insertions were well tolerated, unexpectedly other additions prevented the formation of infectious HSV1 in spite of HSV1-VP26 not being essential. The non-infectious constructs had a very high propensity to form large nuclear aggregates of VP26 and were impaired in nuclear capsid egress, most likely by sequestering other essential proteins into these aggregates. The strain with the least invasive mRFP tag on VP26 induced few nuclear aggregates, and fewer impediments of nuclear egress, secondary envelopment and virion formation. It therefore provides a better backbone to construct new dual-color HSV1(17^+^) variants with other FPs on tegument or glycoproteins. On the other hand, tags that promote mild VP26 aggregate formation may be used to identify further functional interactions with other structural HSV1 proteins by screening for additional synthetic lethal or rescuing mutations [45], [46]. Our data furthermore imply that if compounds could be identified that induce SCP aggregation and thus prevent virion formation; even non-essential viral proteins could provide targets for antiviral therapy.

## Materials and Methods

### Ethics Statement

For immunofluorescence labeling (see below) we applied human serum of healthy, HSV1-seronegative volunteers. This procedure was approved by the Institution Review Board (*Ethikkommision* of Hannover Medical School, approval number 893). Written informed consent of the blood donors, according to the Institution Review Boards, was obtained.

### Cells, Viruses and Antibodies

BHK-21 (ATCC CCL-10) and Vero (ATCC CCL-81) cells were cultured in Minimum Essential Medium (MEM) with 10% and 7.5% fetal calf serum, respectively. Retinal pigment epithelial (RPE) cells (ATCC CRL-4000) were grown in D-MEM/HAM F-12 1:1 with 10% fetal calf serum. HSV1 was propagated in BHK cells and purified as described previously [5], [47]. For this study, we used virions pelleted from the medium of infected cells. The strains HSV1(17^+^) and HSV1(KOS)-GFPVP26_Δaa1–7_ (HSV1-K26GFP) were kindly provided by John Subak-Sharpe (MRC Virology Unit, Glasgow, UK) and Prashant Desai (Johns Hopkins University, Baltimore, MD), respectively [42], [48]. All virus stocks were titrated on Vero cells [49]. Rabbit polyclonal antibodies (PAb) directed against empty capsids (α-LC; [50]), VP26_aa95–112_ [20], VP5 (α-NC-1; [50]), VP22a (α-NC-3,4; [50]), VP23 (α-NC-5; [50]), pUL25 (ID1, R8–3; [51], [52]), pUL36_aa1408–2112_ (#147; [13], [41]), pUL36_aa3048–3057_ (C-term; [53]), pUS3 [54], pUL34 [55], or human PML protein_aa157–394_ (H-238; Santa Cruz Biotechnology Inc., Santa Cruz, CA), as well as the mouse monoclonal antibodies (MAb) against VP5 (5C10, 8F5; [56]; LP12; [57]; H1.4 [Biodesign International, Saco, ME]), VP23 (1D2; [58]), pUL25 and pUL17 (#166, #203; [59]), or ICP8 (HB8180, ATCC, Rockville, MD, USA; provided by Regine Heilbronn, Charité-UniversitΔtsmedizin, Berlin, Germany) were used.

### Construction of HSV1 Strains with Tagged VP26

In our previously described strains HSV1(17^+^)blueLox-mRFPVP26 and -GFPVP26, the fluorescent protein sequences had been inserted into the VP26 sequence by deleting a 65 bp sequence including the first seven VP26 codons for Met-Ala-Val-Pro-Glu-Phe-His (c.f. Fig. 1B; [8]; P. Desai, personal communication). Therefore, in the present study they are called HSV1(17^+^)blueLox-mRFPVP26_Δaa**1**–7_ and -GFPVP26_Δaa**1**–7_. In HSV1(17^+^)blueLox-GFPVP26_Δaa**1**–7_-gDmRFP, glycoprotein D has also been labeled with monomeric RFP at its C-terminus; this virus has been named HSV1(17^+^)blueLox-GFPVP26-gDmRFP previously [8]. To construct additional HSV1 strains in which aa 5 to 7 (Glu-Phe-His) of VP26 were replaced by the fluorescent protein sequence as originally proposed by Desai & Person (1998), first an *Xho*I site was inserted into the ORF UL35. To this end, 500 bp upstream of the insertion site were amplified with *NNN NNN CCT GCA GG* A TGC CCG GCC GAT GAT GG and *NNN NNN CCT GCA GGC TCG AG* T GCG GGA CGG CCA TCG GGA CCG GAG G digested with *Sbf*I and cloned into pUC18 (non-annealing nucleotides in italics, restriction sites underlined; all oligonucleotides were obtained by MWG, Ebersberg, Germany). The 500 bp downstream of the insertion site were amplified with *NNN NNC TCG AG* C CGC CCC AGC ACC GTT ACC ACC GAT AG and *NNN NNN NGG TAC C* CG CCG TGC TGA CCA GCC TAC, digested with *Xho*I and *Kpn*I and cloned into the product of the previous ligation to obtain the plasmid pUC18-UL35.

A linear DNA linker molecule providing an *Nco*I and a *Bsr*GI site was generated by annealing TCG ACC ATG GTG TAC AAC and TCG AGT TGT ACA CCA TGG , and ligated into the *Xho*I site of pUC18-UL35, resulting in pUC18-UL35NB. The fluorescent protein sequences from pEGFP-N1, pEYFP-N1 or pECFP-N1 (Clontech; Mountain View, CA) were excised with *Nco*I and *Bsr*GI and inserted into pUC18-UL35NB to generate pUC18-GFPUL35, -YFPUL35 or -CFPUL35. The complete constructs were amplified from these plasmids with ATG CCC GGC CGA TGA TGG and CGC CGT GCT GAC CAG CCT AC, and Red-recombination was used to replace the *rpsLneo* cassette of the bacterial artificial chromosome (BAC) pHSV1(17^+^)blueLox-ΔVP26 (Fig. 1A; [8]). The resulting BACs were named pHSV1(17^+^)blueLox-GFPVP26_Δaa**5**–7_, -YFPVP26_Δaa**5**–7_, or -CFPVP26_Δaa**5**–7_, respectively (Fig. 1). From these BACs the modified UL35 ORFs were amplified, and the PCR products were sequenced (Seqlab; Göttingen, Germany). For transfection, BAC-DNA was prepared from 500 ml overnight *E. coli* cultures using the NucleoBond™ BAC 100 kit (Macherey & Nagel; Düren, Germany). 5×10^5^ Vero cells in 3.5 cm dishes were grown overnight, transfected with 2 µg BAC-DNA (MBS Mammalian Transfection Kit; Stratagene, La Jolla, CA), and cultured for several days until cytopathic effects developed.

### Immunoblot

Vero cells grown to a density of 5.7×10^5^ per 3.5 cm dish were synchronously infected with 10 plaque forming units (PFU)/cell in 500 µL for 2 h on ice, and then further cultured in regular growth medium at 37°C. After 1 h, the cells were washed for 3 min with citrate buffer (40 mM citric acid, pH 3, 135 mM NaCl, 10 mM KCl) to inactivate any extracellular virions that had not been internalized [60], and returned to fresh medium to initiate a synchronous infection. At different time points, the culture supernatants were collected and titrated. Infected cells and their complete supernatants were also harvested at 48 h post infection (p.i.). Viral particles released from infected cells into the culture medium were pelleted in a Beckman TLA100.3 rotor at 50,000 rpm for 30 min and resuspended in 100 µL hot SDS-PAGE sample buffer [61] with protease inhibitors. The cells were scraped into 200 µL of hot sample buffer with protease inhibitors and sheared by 50 passages through a 24 gauge needle. After SDS-PAGE on linear 5 to 15% polyacrylamide gradient gels and transfer onto a nitrocellulose membrane, viral proteins were probed with specific primary antibodies and alkaline-phosphatase-coupled secondary antibodies (Dianova; Hamburg, Germany).

### Fluorescence Microscopy

Vero cells grown on cover slips to a density of 5 to 10×10^4^ per 2 cm^2^ were synchronously infected as described above at a multiplicity of infection (MOI) of 10 PFU/cell. At 9 h p.i., cells were fixed with 3% (w/v) para-formaldehyde (PFA) in PBS and permeabilized with 0.1% Triton X-100 (TX-100) for 5 min, fixed with absolute methanol, or fixed and permeabilized with PHEMO-fix (3.7% [w/v] PFA, 0.05% [w/v] glutaraldehyde, 0.5% [v/v] TX-100 in PHEMO buffer [68 mM PIPES, 25 mM HEPES, pH 6.9, 15 mM EGTA, 3 mM MgCl_2_, 10% DMSO]) for 10 min. The HSV1 Fc receptor was blocked with 10% (v/v) human serum of healthy, HSV1-seronegative volunteers, and the samples were immunolabeled as described before [5], [8], [47], [49], [62], [63]. All secondary antibodies were highly pre-adsorbed against cross-reactivities against other species than the intended one, and were coupled to lissamine-rhodaminesulfonyl chloride, RedX, fluorescein isothiocyanate, or Cy5 (Dianova, Hamburg, Germany). The cells were analyzed on an Axiovert 200M microscope equipped with a LSM 510 META confocal laser scanning unit (ZEISS, Jena, Germany) using a plan-apochromatic 63× oil-immersion objective with an 1.4 numeric aperture. Image acquisition and processing was performed using the ZEISS LSM imaging software, ImageJ 1.35 (Wayne Rasband; National Institute of Health, USA; http://rsb.info.nih.gov/ij/), and Adobe® Photoshop CS (Adobe Systems, San Jose, CA, USA). For classification and quantification of the intranuclear VP26 phenotype, we used a primary data set obtained from an analysis of at least 85 randomly chosen cells fixed at several time points from 4 to 12 h p.i. [8]. The degree of nuclear aggregate formation was classified based on the density and appearance of the anti-VP26 or GFPVP26 signals into the categories “none”, “single”, “grainy”, and “aggregated”.

### Correlative Light and Electron Microscopy

RPE cells grown to 4×10^5^ cells per 8.5 cm^2^ on glass bottom dishes (MatTek Corporation, Ashland, MA, USA) with self-made marks to aid the localization of individual cells were synchronously infected with HSV1(KOS)-GFPVP26_Δaa**1**–7_ at an MOI of 10 PFU/cell. After 19.5 h of infection, the fluorescence signals were documented with a Zeiss Axiovert 200 microscope prior to fixation for 1 h at room temperature with 2% (w/v) glutaraldehyde in 130 mM cacodylate buffer at pH 7.4 containing 2 mM CaCl_2_ and 10 mM MgCl_2_. The cells were washed and further fixed and contrasted with 1% (w/v) OsO_4_ in 165 mM cacodylate buffer at pH 7.4 containing 1.5% (w/v) K_3_Fe^III^(CN)_6_ for 1 h followed by incubation in 0.5% (w/v) uranyl acetate in 50% (v/v) ethanol overnight. The cells were flat-embedded in Epon, and semi-thin sections cut parallel to the substrate were used to identify cells characterized by a high amount of nuclear aggregates and an indicative nuclear morphology that had been documented by epifluorescence microscopy prior to fixation. Such cells were then relocated in ultrathin sections of 50 nm and analyzed with a FEI Tecnai G2 T20 electron microscope. We analyzed ultrathin sections of more than 50 cells, and furthermore in detail by correlative light and electron microscopy 8 cells each containing more than 5 nuclear aggregates.

## Results

### Construction of HSV1 Strains with Tagged VP26

To identify strains whose capsid formation and intracellular trafficking mimic that of their untagged parent, we compared several constructs with different FP tags on VP26 that we had introduced into the HSV1(17^+^)blueLox BAC. The HSV1(17^+^)blueLox-GFPVP26_Δaa**1**–7_ strain, as the previously reported HSV1(KOS)-GFPVP26_Δaa**1**–7_, lacks 65 bp upstream of the start codon of GFPVP26 including the seven N-terminal aa of VP26 (Fig. 1; P. Desai, personal communication and data not shown; [8], [42]). Both strains propagate to similar titers in BHK cells [8].

In this study, we have generated the new strains HSV1(17^+^)blueLox-CFPVP26_Δaa**5–**7_, -GFPVP26_Δaa**5**–7_, and -YFPVP26_Δaa**5**–7_ (c.f. Fig. 1A, B) according to a strategy originally described by Desai & Person (1998). The codons for aa 5 to 7 of VP26 were replaced by different FPs with the linkers His-Ser-Thr and Asn-Ser-Ser at their respective N- and C-termini (Fig. 1B). The viral genomes of these strains contained the engineered changes in UL35 (arrowheads in Fig. 2A) but not any major unwanted alterations as shown by restriction enzyme digestion with *Not*I, *Eco*RI, *Eco*RV or *Sbf*I (Fig. 2A and data not shown). There was some heterogeneity among strains in the *Not*I joint fragments around 3.3 kb (asterisks in Fig. 2A) that do not seem to have any influence on infectivity as reported previously [8], [63], [64]. Sequencing the UL35 region of the BACs confirmed the correct insertion of the FP sequences (not shown). But after transfection of Vero cells with these BACs, unexpectedly only pHSV1(17^+^)blueLox-CFPVP26_Δaa**5**–7_ gave rise to regular fluorescent plaques, whereas -GFPVP26_Δaa**5**–7_ yielded tiny plaques, and -YFPVP26_Δaa**5**–7_ merely single fluorescent cells (data not shown). So, surprisingly the four additional aa derived from the authentic VP26 N-terminus, the three linker aa His-Ser-Thr or maintaining the authentic non-coding 50 bp upstream sequences inhibited replication of pHSV1(17^+^)blueLox-GFPVP26_Δaa**5**–7_ and -YFPVP26_Δaa**5**–7_, but not that of pHSV1(17^+^)blueLox-CFPVP26_Δaa**5**–7_.

To further characterize potential differences between these strains, we compared their growth kinetics and protein expression. While HSV1(17^+^)blueLox-ΔVP26 and -mRFPVP26_Δaa**1**–7_ were attenuated in BHK cells [8], there was no growth difference between HSV1(17^+^)blueLox, -ΔVP26 and -mRFPVP26_Δaa**1**–7_ in Vero cells (Fig. 2B). But compared to those, -CFPVP26_Δaa**5–**7_ and -GFPVP26_Δaa**1**–7_ were slightly attenuated, and -GFPVP26_Δaa**1**–7_ replicated with delayed kinetics (Fig. 2B). Likewise, HSV1(17^+^)blueLox-GFPVP26_Δaa**1**–7_ is delayed in BHK cells when compared to -mRFPVP26_Δaa**1**–7_ [8]. Thus, the growth properties of these HSV1 strains varied depending on the type of the tag, the location of the tag, and the cell line. After synchronous infection of Vero cells with an MOI of 10 PFU/cell (Fig. 2C), the parental HSV1(17^+^) synthesized more proteins and secreted more virions into the medium than HSV1(17^+^)blueLox and its derivatives. An antibody raised against VP26 detected a band around 12 kDa after infection with HSV1(17^+^) or HSV1(17^+^)blueLox but not with HSV1(17^+^)blueLox-ΔVP26. After infection with the tagged strains, there was one major band at 38 kDa for -CFPVP26_Δaa**5**–7_ or -GFPVP26_Δaa**1**–7_ in addition to some other minor bands. mRFPVP26_Δaa**1**–7_ had a higher apparent molecular weight, although mRFP is about 1 kDa smaller than GFP. More of the CFPVP26_Δaa**5**–7_ fusion protein was synthesized when compared to GFPVP26_Δaa**1–**7_ or mRFPVP26_Δaa**1**–7_, possibly due to upstream promoter elements that were missing in the latter two strains (c.f. Fig. 1B). These data show furthermore that all VP26 fusion proteins of the infectious HSV1 strains were incorporated into secreted virions as full-length proteins.

### Nuclear Aggregates Induced by HSV1-XFPVP26 Impair Nuclear Capsid Egress

**Figure 3:**
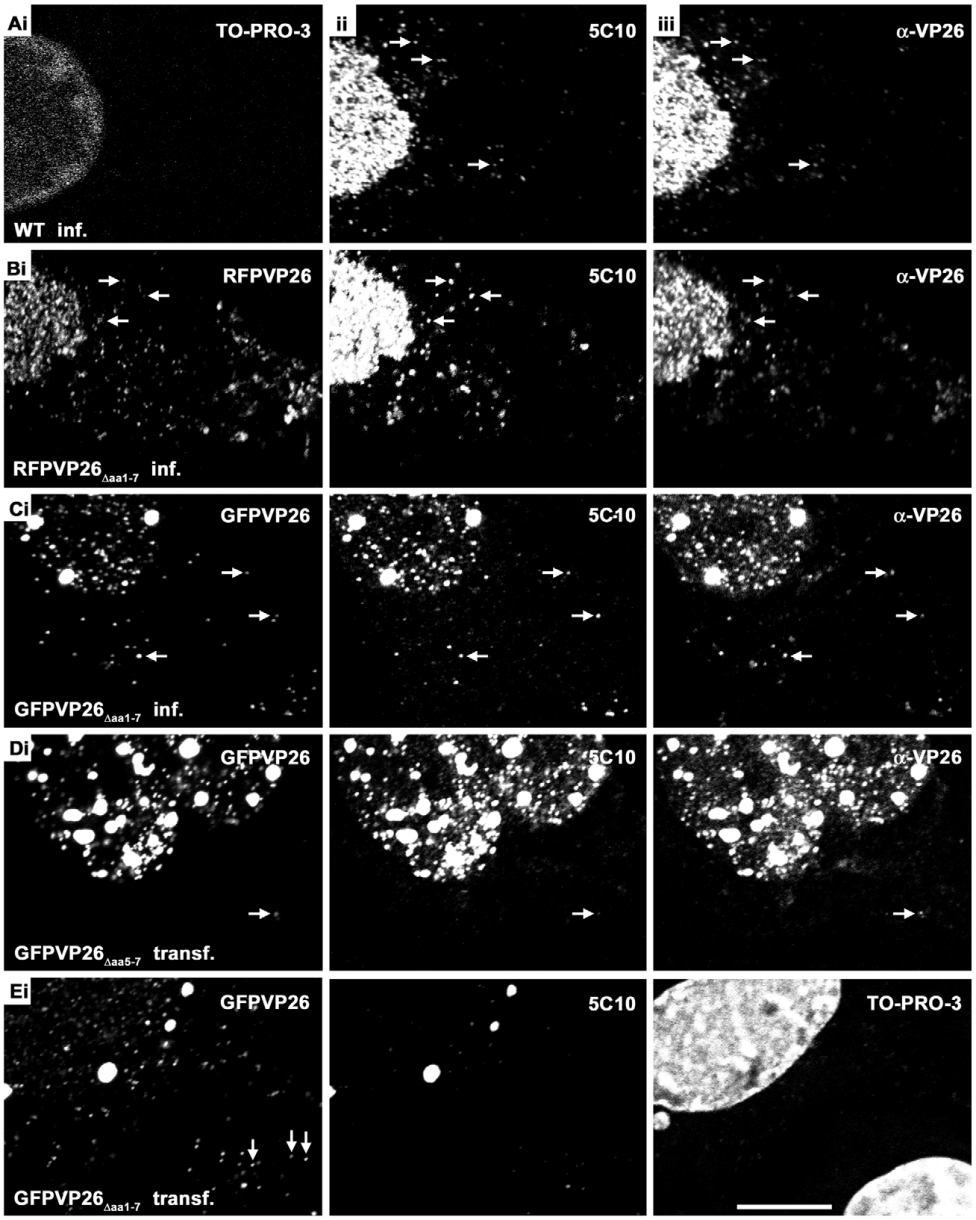
Nuclear aggregates induced by HSV1-XFPVP26 impair nuclear capsid egress. Vero cells were infected (inf.) with 10 PFU/cell of HSV1(17^+^)blueLox (A, wild type), HSV1(17^+^)blueLox-mRFPVP26_Δaa**1**–7_ (B), or HSV1(17^+^)blueLox-GFPVP26_Δaa**1**–7_(C), and fixed at 9 h with PFA. Alternatively, cells were transfected (transf.) with pHSV1(17^+^)blueLox-GFPVP26_Δaa**5**–7_ (D) or pHSV1(17^+^)blueLox-GFPVP26_Δaa**1**–7_ (E), and fixed at 24 h. In addition to the intrinsic fluorescence of the XFPVP26 constructs (mRFPVP26 or GFPVP26), the subcellular localization of VP26 (α-VP26) and VP5 (MAb 5C10) were analyzed after permeabilization with TX-100 and immunolabeling by confocal fluorescence microscopy. The nuclei were stained with TO-PRO-3 (A, E). Arrows highlight cytoplasmic capsids (A–D) or incoming capsids at the nuclear rim of a neighboring cell (Ei). Scale bar: 10 µm.

To assess the influence of VP26 tagging on virus assembly, we infected Vero cells with 10 PFU/cell for 9 h, by which time many wild type capsids have left the nucleus but not the cells [8], [63], and analyzed them by confocal fluorescence microscopy. After infection with HSV1(17^+^)blueLox (Fig. 3A, wild type), -mRFPVP26_Δaa**1**–7_ (Fig. 3B), or -GFPVP26_Δaa**1**–7_ (Fig. 3C), nuclear and cytoplasmic capsids labeled by antibodies against VP26 or VP5 were also highlighted by mRFP or GFP in the respective strains. The signals for nuclear mRFPVP26_Δaa**1**–7_ and GFPVP26_Δaa**1**–7_ were similar or weaker than those of anti-VP5 (Fig. 3B/Cii) and anti-VP26 (Fig. 3B/Ciii). In contrast, more cytoplasmic capsids (arrows in Fig. 3A–C) were detected by mRFPVP26_Δaa1–7_ (Fig. 3Bi) or GFPVP26_Δaa**1**–7_ (Fig. 3Ci) than by the antibodies. However, HSV1(17^+^)blueLox-GFPVP26_Δaa**1**–7_ but not -mRFPVP26_Δaa**1**–7_ or HSV1 wild type also induced the formation of large nuclear GFP aggregates that also contained VP5 and VP26 (Fig. 3C). Furthermore, there were fewer cytoplasmic capsids after infection with HSV1(17^+^)blueLox-GFPVP26_Δaa**1**–7_ than with -mRFPVP26_Δaa**1**–7_. Later during infection, HSV1(17^+^)blueLox and -mRFPVP26_Δaa**1**–7_ also induced such nuclear aggregates (not shown). Notably, in other cell lines such as HeLa cells, RPE cells or primary neurons, the nuclear aggregates of HSV1(17^+^)blueLox-GFPVP26_Δaa**1**–7_ were even more prominent than in Vero cells, but -mRFPVP26_Δaa**1**–7_ always induced fewer aggregates (not shown).

As described above, transfection with the BACs pHSV1(17^+^)blueLox-GFPVP26_Δaa**5**–7_ or -YFPVP26_Δaa**5**–7_ did not yield virions. But an analysis of transfected cells revealed small nuclear GFPVP26_Δaa**5**–7_ flecks of the typical capsid size as well as large nuclear GFPVP26_Δaa**5**–7_ aggregates, which were both also labeled by antibodies against VP5 or VP26 (Fig. 3D). However, there were much fewer cytoplasmic GFPVP26_Δaa**5**–7_ capsids than after infection with HSV1(17^+^)blueLox-GFPVP26_Δaa**1**–7_ (c.f. Fig. 3C with Fig. 3D). The nuclear aggregates after transfection with -YFPVP26_Δaa**5**–7_ were even larger and of irregular shape, and there were no individual nuclear or cytoplasmic capsids (data not shown). In contrast, the strain HSV1(17^+^)blueLox-CFPVP26_Δaa**5**–7_ induced fewer and smaller nuclear aggregates, and there were single nuclear and cytoplasmic capsids, both after transfection and infection (not shown). The difference between HSV1(17^+^)blueLox-GFPVP26_Δaa**1**–7_ and -GFPVP26_Δaa**5**–7_ might have been due to different expression levels as the protein GFPVP26_Δaa**1**–7_ had been expressed from viral genomes, whereas the mRNAs for GFPVP26_Δaa**5**–7_ were transcribed from the transfected BAC plasmids. However, in the case of HSV1(17^+^)blueLox-GFPVP26_Δaa**1**–7_, capsids were exported to the cytoplasm after both, infection for 9 h (Fig. 3C), or transfection for 24 h (Fig. 3E). While GFPVP26_Δaa**5**–7_ was largely confined to the nucleus (Fig. 3Di), GFPVP26_Δaa**1**–7_ particles even spread from the transfected to neighboring cells and were transported to the neighboring nucleus (Fig. 3Ei, white arrows). After PHEMO fixation, DNA could be detected in the nuclear aggregates by TO-PRO-3 staining, whereas after PFA fixation this was not the case (Fig. 3Eiii) as described previously [4].

**Figure 4:**
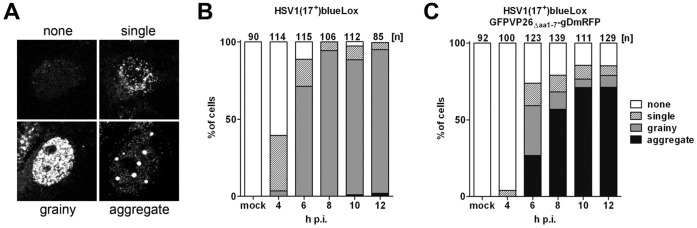
Quantification of nuclear aggregate formation. Vero cells were infected with 10 PFU/cell of HSV1(17^+^)blueLox (B) or HSV1(17^+^)blueLox-GFPVP26_Δaa**1**–7_-gDmRFP (C), and the cells were fixed at 4, 6, 8, 10, or 12 h. After permeabilization, HSV1(17^+^)blueLox infected cells were labeled with an antibody directed against VP26. According to their intranuclear VP26 phenotype, the cells were classified into “none”, “single”, “grainy” and “aggregate” (A). The numbers above the columns describe the number of nuclei analyzed for each time point.

We have reported previously that the capsids of HSV1(17^+^)blueLox accumulate faster in the cytoplasm than those of HSV1(17^+^)blueLox-GFPVP26_Δaa**1**–7_-gDmRFP (Fig. 8C in [8]). Here, we have analyzed also the time course of nuclear aggregate formation using the same data set of randomly collected confocal fluorescence microscopy images. The cells were classified by their phenotypes as having (i) no nuclear VP26 as in uninfected cells (none), (ii) individual nuclear capsids (single), (iii) many capsids filling the entire nucleus (grainy), or (iv) nuclear aggregates containing VP26 (Fig. 4A). Already 4 h after infection with HSV1(17^+^)blueLox (Fig. 4B), more than 30% of the cells contained nuclear capsids, and by 8 h almost 90% of the nuclei contained many nuclear capsids. By 12 h, the first nuclear aggregates appeared in the nuclei of some few cells. In contrast, after infection with HSV1(17^+^)blueLox-GFPVP26_Δaa**1**–7_-gDmRFP (Fig. 4C), there were already prominent nuclear aggregates in 25% of the nuclei after 6 h, and by 10 h this had risen to 70% [8]. In summary, our data indicate an inverse correlation between the amount of nuclear aggregate formation and the efficiency of nuclear capsid egress and thus infectivity. The extent of inhibition depended on the type of the tag with mRFP or CFP being almost non-invasive, on the location of the tag with XFPVP26_Δaa**1**–7_ being better tolerated than XFPVP26_Δaa**5**–7_, and on the cell line with Vero cells being less prone to aggregate formation than HeLa, RPE or neuronal cells.

### Nuclear Aggregates Contain Capsid Proteins but not Capsids

**Figure 5:**
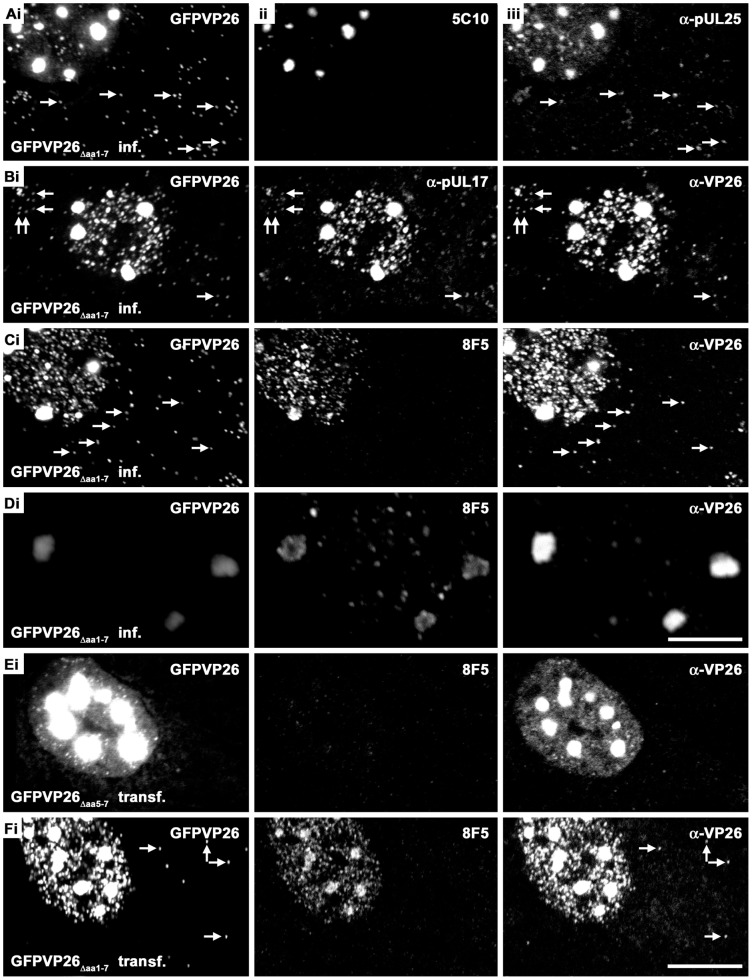
Nuclear aggregates contain capsid proteins. Vero cells were infected (inf.) with 10 PFU/cell of HSV1(17^+^)blueLox-GFPVP26_Δaa**1**–7_ for 9 h (A–D) or transfected (transf.) with pHSV1(17^+^)blueLox-GFPVP26_Δaa**5**–7_ (E) or pHSV1(17^+^)blueLox-GFPVP26_Δaa**1**–7_ (F) for 24 h. The cells were fixed with PFA and permeabilized with TX-100. GFPVP26 was detected by its intrinsic fluorescence (left column). Furthermore, the cells were labeled with different VP5 antibodies (MAb 8F5 or 5C10) and with α-VP26, α-pUL25 or α-pUL17, and analyzed by confocal fluorescence microscopy. For row D, the specimen was scanned at higher magnification and lower photomultiplier settings. The arrows point to cytoplasmic capsids. Bars: 5 µm (D), 10 µm (F).

To further characterize the protein composition of the aggregates, we determined the subcellular localization of other HSV1 proteins in infected or transfected Vero cells (summarized in Table 1). Antibodies against two minor capsid associated proteins, pUL25 (Fig. 5Aiii) or pUL17 (Fig. 5Bii), as well as VP26 (Fig. 5Biii, 5Ciii, 5Diii), co-localized with the XFPVP26 throughout the nuclear aggregates, both after infection with HSV1(17^+^)blueLox-GFPVP26_Δaa**1**–7_ (Fig. 5A-D), or after transfection with pHSV1(17^+^)blueLox-GFPVP26_Δaa**5**–7_ (Fig. 5E). The MAb 5C10 directed against mature hexon epitopes also highlighted the entire volume of the aggregates(Fig. 5Aii, 3Cii, 3Dii, 3Eii), while MAb 8F5, also recognizing mature hexon epitopes, was enriched on the periphery of the aggregates (Fig. 5Cii, 5Dii, 5Fii). Since de Oliveira et al. (2008) detected a high amount of XFPVP26 on the periphery of the aggregates [4], we also imaged them with less sensitive detection settings at higher magnification (Fig. 5D). Occasionally, large aggregates appeared to consist of smaller entities but the GFPVP26 or anti-VP26 signals did not seem to be particularly enriched on the rims of the aggregates (Fig. 5Di and 5Diii). Nevertheless, MAb LP12 and MAb H1.4, two other antibodies directed against VP5, also preferentially labeled the periphery but not the center of most aggregates (not shown; summarized in Table 1). Surprisingly, in cells transfected with pHSV1(17^+^)blueLox-GFPVP26_Δaa**5**–7_, the mature hexon epitope recognized by MAb 8F5 was not detected at all (Fig. 5Eii), whereas the other antibodies directed against VP5 resulted in similar patterns as after infection with HSV1(17^+^)blueLox-GFPVP26_Δaa**1**–7_ (summarized in Table 1). This was not due to different expression levels in infected or transfected cells, as the MAb 8F5 epitope was formed after both, infection or transfection with pHSV1(17^+^)blueLox-GFPVP26_Δaa**1**–7_ (Fig. 5Fii).

**Table 1:** Characterization of HSV1-GFPVP26 nuclear aggregates.

Antigen	Antibody	HSV1(17^+^)blueLox- GFPVP26	Subcellular localization	Detection in nuclear aggregates	
**VP26**	pAb aa 95–112	Δaa**1**–7 infection	mainly nuclear,	+++	entire
		Δaa**5**–7 transfection	cytoplasmic dots		
**VP5**	mAb5C10	Δaa**1**–7 infection	mainly nuclear,	+++	entire
		Δaa**5**–7 transfection	cytoplasmic dots		
	mAb 8F5	Δaa**1**–7 infection	mainly nuclear,	++	periphery
		Δaa**1**–7 transfection	cytoplasmic dots		
		Δaa**5**–7 transfection	epitope not formed	–	–
	mAb H1.4	Δaa**1**–7 infection	mainly nuclear,	++	periphery
		Δaa**5**–7 transfection	cytoplasmic dots		
	mAb LP12	Δaa**1**–7 infection	mainly nuclear,	++	periphery
		Δaa**5**–7 transfection	cytoplasmic dots		
**VP22a**	pAb NC-3,4	Δaa**1**–7 infection	mainly nuclear,	- (PFA)	periphery
			cytoplasmic dots	+++ (MeOH)	
				+ (PHEMO)	
		Δaa**5**–7 transfection		++ (PHEMO)	periphery
**VP23**	pAb NC-5	Δaa**1**–7 infection	nuclear, nuclear rim,	–	–
		Δaa**5**–7 transfection	perinuclear region		
	mAb 1D2	Δaa**1**–7 infection	nuclear rim	–	–
		Δaa**5**–7 transfection			
**pUL25**	mAb ID1,	Δaa**1**–7 infection	mainly nuclear,	+++	entire
	pAb#166	Δaa**5**–7 transfection	cytoplasmic dots		
**pUL17**	pAb #203	Δaa**1**–7 infection	mainly nuclear,	+++	entire
	aa 154-703	Δaa**5**–7 transfection	cytoplasmic dots		
**light capsid**	pAb anti-LC	Δaa**1**–7 infection	mainly nuclear,	+++	periphery
		Δaa**5**–7 transfection	cytoplasmic dots		
**pUL36**	pAb #147	Δaa**1**–7 infection	mainly perinuclear,	–	–
	aa 1408–2112	Δaa**5**–7 transfection	cytoplasm	+	periphery
	pAb C-term	Δaa**1**–7 infection	mainly perinuclear,	–	–
	aa 3048–3057	Δaa**5**–7 transfection	cytoplasm	+	periphery
**pUS3**	pAb aa 98–364	Δaa**1**–7 infection	nuclear, nuclear rim,	–	–
		Δaa**5**–7 transfection	perinuclear region		
**pUL34**	pAb aa 1–252	Δaa**1**–7 infection	mainly nuclear rim	–	–
		Δaa**5**–7 transfection			
**ICP8**	mAb HB8180	Δaa**1**–7 infection	nuclear	–	–
		Δaa**5**–7 transfection			
**PML**	pAb H-238	Δaa**1**–7 infection	degraded	–	–
	aa157–394	Δaa**5**–7 transfection			

Vero cells were infected with HSV1(17^+^)blueLox-GFPVP26_Daa**1**–7_ at an MOI of 10 PFU/cell and fixed at 9 h, or transfected with pHSV1(17^+^)blueLox-GFPVP26_Daa**5**–7_ or -GFPVP26_Daa**1**–7_ and fixed at 24 h. The cells had been fixed with PFA unless indicated otherwise, and subsequently labeled with different antibodies. MeOH, methanol fixation; PHEMO, PHEMO fixation; –, not detected.

**Figure 6:**
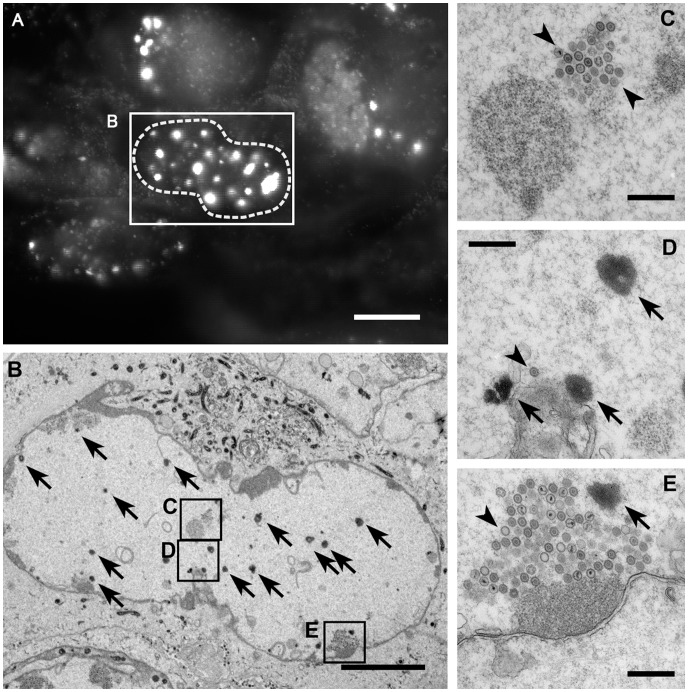
Nuclear aggregates do not contain capsids. RPE cells were infected with HSV1(KOS)-GFPVP26_Δaa**1**–7_ at an MOI of 10 PFU/cell. At 19.5 h, the cells were analyzed by fluorescence microscopy to identify nuclei with bright fluorescence spots (A). After fixation, embedding and sectioning these cells were further analyzed by electron microscopy (B–E). The nuclei contain nuclear capsids (arrowheads) as well as the amorphous electron dense material representing the aggregates (arrows) identified by fluorescence microscopy. The aggregates do not contain capsids (arrowheads). Bars: 10 µm (A), 5 µm (B), and 500 nm (C–E).

Using correlative light and electron microscopy, we further analyzed RPE cells after infection with HSV1(KOS)-GFPVP26_Δaa**1**–7_ (Fig. 6), a virus-cell combination that was particularly prone to nuclear aggregate formation. Prior to fixation, the subcellular localization of the nuclear aggregates was documented by fluorescence microscopy (Fig. 6A). Corresponding electron micrographs of ultrathin sections through such cells showed (Fig. 6B), particularly at higher magnifications (Fig. 6C–E) that these aggregates contained amorphous electron dense material (arrows) but no capsids, and that capsids (arrowheads) were found rarely at the periphery of the aggregates. The aggregates reacted specifically with the uranyl acetate stain that is applied after sectioning for contrast, indicating that this material was rich in proteins and/or nucleic acids (data not shown). The different types of A, B, and C capsids had all been generated, and sometimes also clustered into larger assemblies (Fig. 6C and E, arrowheads). In summary, immune labeling, the TO-PRO-3 staining after PHEMO fixation (not shown), and the electron microscopy analysis demonstrated that the nuclear aggregates comprised most, if not all capsid proteins and DNA, but many of them only abut HSV1 capsids, and the MAb 8F5 epitope was only formed on the aggregate periphery, or not at all after transfection with pHSV1-GFPVP26_Δaa**5**–7_.

### Nuclear Aggregates do not Sequester pUL36, pUS3 or pUL34 and are Neither Replication Compartments nor Promyelocytic Leukemia Protein Nuclear Bodies

**Figure 7:**
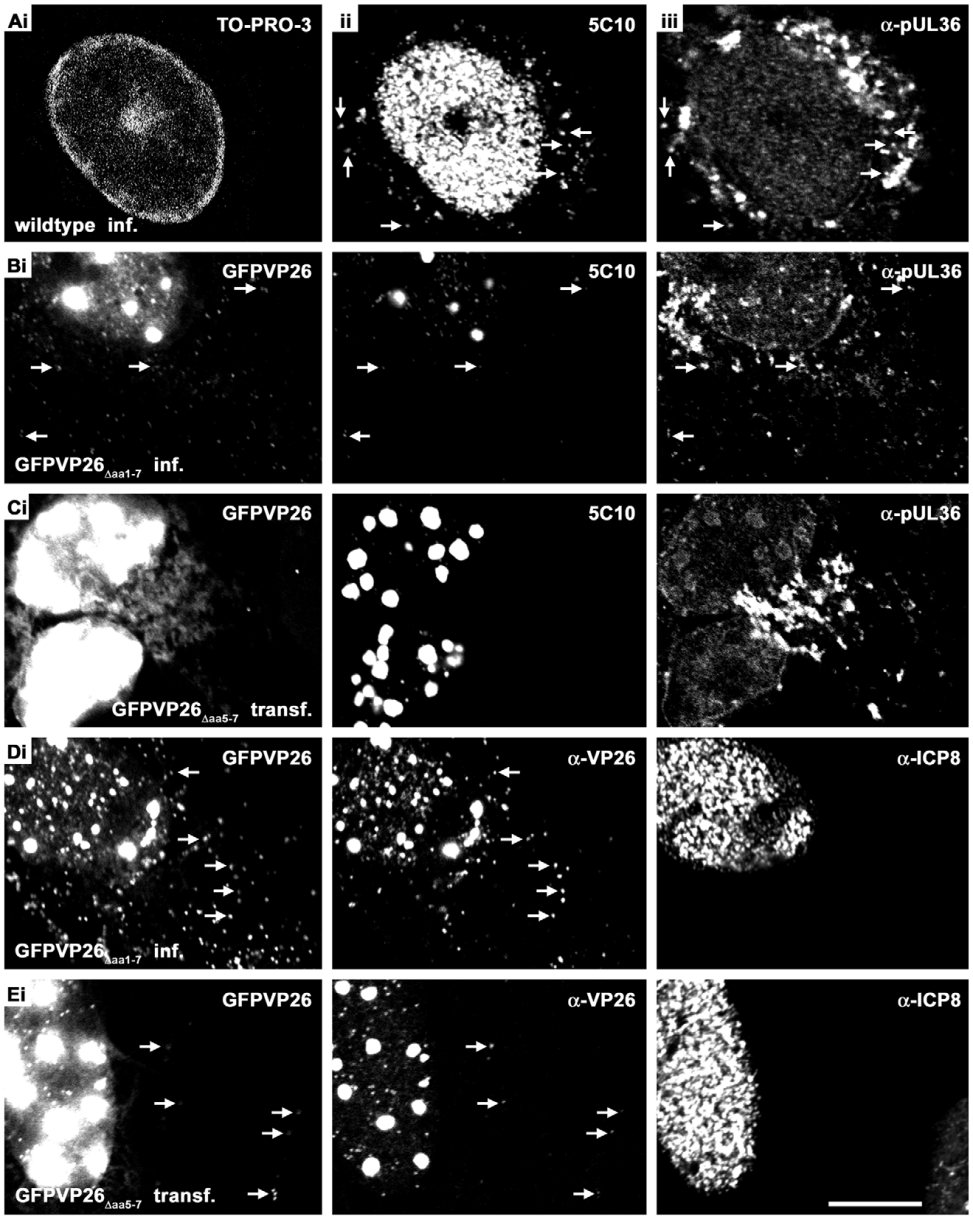
Nuclear GFPVP26 aggregates do not sequester pUL36 and are not DNA replication compartments. Vero cells were infected (inf.) for 9 h with 10 PFU/cell of HSV1(17^+^)blueLox (A) or HSV1(17^+^)blueLox-GFPVP26_Δaa**1**–7_ (B, D), or transfected (transf.) with the pHSV1(17^+^)blueLox-GFPVP26_Δaa**5**–7_ for 24 h(C, E). The cells were fixed with PFA and permeabilized with TX-100. GFPVP26 was detected by its intrinsic fluorescence. Furthermore, the cells were labeled with anti-pUL36_aa1408–2112_ and with MAb 5C10 against VP5 (A–C), against the single-strand DNA binding protein ICP8 and VP26 (D, E), or with TO-PRO-3 to stain the DNA (A). The arrows point to cytoplasmic capsids. Scale bar: 10 µm.

Like pUL17 and pUL25, the large inner tegument protein pUL36 has also been implicated in nuclear capsid egress [65]–[67]. After infection with HSV1(17^+^)blueLox (Fig. 7A) or HSV1(17^+^)blueLox-GFPVP26_Δaa**1**–7_ (Fig. 7B), pUL36 as detected by antibodies raised against a middle region (pUL36_aa1408–2112_) or its C-terminus (pUL36_aa3048–3057_; not shown) was enriched in the perinuclear cytoplasm and localized in the cell periphery, where it co-localized with VP5 (arrows in Fig. 7Aii and 7Aiii), as reported previously [63]. The host chromatin, stained with TO-PRO-3, was marginalized to the nuclear periphery (Fig. 7Ai), as reported previously [68]. pUL36 did not colocalize with nuclear aggregates after infection with HSV1(17^+^)blueLox-GFPVP26_Δaa**1**–7_ (7Biii). After transfection with pHSV1(17^+^)blueLox-GFPVP26_Δaa**5**–7_ that does not release capsids from the nucleus into the cytoplasm (Fig. 7Ci), pUL36 was similarly distributed in the cytoplasm (Fig. 7Ciii). In few cells transfected with pHSV1(17^+^)blueLox-GFPVP26_Δaa**5**–7_, pUL36 was enriched on the periphery of the nuclear aggregates (Fig. 7Ciii). In contrast, the HSV1 protein kinase pUS3 and the integral membrane protein pUL34 that function in nuclear capsid egress (reviewed in [66], [67] were localized at the nuclear envelope as after infection with HSV1 wild type, and not redirected to the nuclear aggregates (not shown).

The morphology of the nuclear aggregates seemed to have some resemblance to nuclear DNA replication compartments [69]–[71]. After infection with HSV1(17^+^)blueLox, the single-strand DNA binding protein ICP8, an essential component of the HSV1 replication machinery, and GFPVP26 occupied very similar nuclear regions as reported previously [72]. Higher resolution showed that ICP8 and capsid proteins did not co-localize although they were located very close to each other (not shown). Also after infection with HSV1(17^+^)blueLox-GFPVP26_Δaa**1**–7_ (Fig. 7D) or transfection with pHSV1(17^+^)blueLox-GFPVP26_Δaa**5**–7_ (Fig. 7E), ICP8 was not targeted to the nuclear aggregates. The SCP of VZV interacts with a promyelocytic leukemia protein, a component of nuclear promyelocytic leukemia protein bodies that sequester progeny VZV capsids and thus reduce virion formation [73], [74]. We therefore tested whether the nuclear HSV1 aggregates or capsids might have been trapped by this intrinsic antiviral defense mechanism. However, while uninfected cells contain several ND10 domains in each nucleus, the promyelocytic leukemia protein bodies were degraded after infection with HSV1(17^+^)blueLox-GFPVP26_Δaa**1**–7_ as well as with HSV1(17^+^)blueLox (not shown), as reported before for HSV1 wild type [75]. Therefore, the nuclear aggregates do not represent anti-viral promyelocytic leukemia protein bodies.

## Discussion

### Tagging of HSV1-VP26

Our attempts to tag the N-terminus of the non-essential small capsid protein VP26 of HSV1(17^+^)blueLox revealed that subtle differences in the FP domain defined whether a transfected BAC replicated with wild type kinetics or was non-infectious (c.f. Fig. 1). The degree of nuclear aggregate formation correlated with an impediment of nuclear capsid egress and depended on the presence of upstream non-coding sequences, the tag with mRFP or CFP being less invasive than GFP or YFP, the location of the tag with XFPVP26_Δaa**1**–7_ being better tolerated than XFPVP26_Δaa**5**–7_, and the cell type with Vero cells generating fewer aggregates than HeLa, RPE or primary neurons.

In cells infected with HSV1(17^+^), immunolabeling for VP5 or VP26 does not reveal such nuclear aggregates up to 15 h p.i., but rather results in a grainy pattern that most likely represents individual capsids (Fig. 3A; 4B; 7A; [62], [63]). Notably, release of infectious particles from the infected cells commences as early as 12 h p.i. (Fig. 2B; [8]). Therefore, we consider the nuclear VP26 aggregates that formed with certain FP tags as early as 6 h p.i. to be non-physiological. Since the subcellular distribution of mRFPVP26_Δaa**1**–7_ resembles that of the authentic VP26 more closely than the GFP variants, HSV1-mRFPVP26_Δaa**1**–7_ strain seems best suited to investigate the assembly and intracellular trafficking of HSV1.

The distinct phenotypes of the FPVP26 constructs may be related to differences in their quaternary structure. While GFP and YFP tend to dimerize with dissociation constants around 0.1 mM, the N146I mutation in CFP does not only increase its brightness but also its dissociation constant to 3 mM [76]–[79]. mRFP is not related to GFP and has been designed to remain monomeric [80]. Thus, dimerization and aggregate induction of GFPVP26_Δaa**5**–7_ and YFPVP26_Δaa**5**–7_ may be favored when compared to CFPVP26_Δaa**5**–7_ and mRFPVP26_Δaa**1**–7_, given the high concentration of capsid proteins in the nucleoplasm, and given an intrinsic dissociation constant of 0.02 mM for recombinant VP26 [28]. Furthermore, deleting non-coding sequences upstream of the UL35 gene reduced expression, and thus possibly the toxicity of GFPVP26_Δaa**1**–7_ when compared to GFPVP26_Δaa**5**–7_. Indeed, these 50 bp encompass a sequence with perfect consensus to the Inr element YYANWYY required for efficient transcription from HSV1 late promoters [81]. Another similar sequence within the deletion and two other sequences upstream of the deletion diverge from the Inr consensus in the last base pair (Fig. 1B).

In a triple-color HSV1(F) strain, VP26 has been tagged at codon 2 with YFP^VenusA206K^, with this YFP having a mutation that also increases its dissociation constant from 0.11 mM to 74 mM [11], [77], [78], [82]. This strain is indeed infectious in contrast to our HSV1(17^+^)blueLox-YFPVP26_Δaa**5**–7_, but HSV1(F)-YFP^VenusA206K^VP26_Δa1_ also replicates less well than its parental strain, and induces nuclear aggregates even in Vero cells [11]. An HSV1(F) strain with N-terminal mRFP on VP26 lacking only 4 initial codons but not the authentic non-coding upstream sequences is also infectious; however, in contrast to our HSV1(17^+^)blueLox-mRFPVP26_Δaa**1**–7_ reported here, this strain also induces nuclear aggregates late in infection, and it is a bit attenuated in Vero cells, too [4]. Furthermore, a PrV-mRFPVP26 strain with authentic upstream sequences and mRFP located between codon 2 and 3 also induces nuclear aggregates and forms smaller plaques [44], [83].

Thus, it seems more likely that the reduced protein expression due to 50 upstream bp missing, and not the removal of the 4 N-terminal codons contributes to the almost wild type properties of our HSV1(17^+^)blueLox-mRFPVP26_Δaa1–7_. We considered that further compensatory mutations might have allowed efficient capsid assembly and nuclear egress of some XFPVP26 mutants. However, when we monitored several independent lots of cells transfected with BAC DNA, there were no signs of any adaption periods during which CFPVP26_Δaa5–7_, mRFPVP26_Δaa1–7_ or GFPVP26_Δaa1–7_ changed from initial slower growth to faster replication cycles. Furthermore, all experiments were conducted with virions of passage 2 or 3 post transfection, and GFPVP26_Δaa5–7_ and YFPVP26_Δaa5–7_ did not replicate even after prolonged culture. In summary these studies suggest that VP26 can be successfully tagged with FP domains while maintaining infectivity and efficient nuclear capsid egress when the extent of nuclear aggregate formation is kept low.

### Nuclear Aggregates do not Contain Capsids

In contrast to de Oliveira et al. (2008), we could detect the tagged VP26, VP5 (MAb 5C10), pUL17 and pUL25 not only on the rim but also within the nuclear aggregates. The aggregates resemble so-called assemblons that can form with HSV1(F) as early as 6 h but become prominent at 16 h of infection, and that also contain the capsid proteins VP5 and VP19c [84], [85]. Ward et al. (1996) therefore suggested that assemblons represent the nuclear site of capsid assembly [84]. Consistent with this hypothesis the nuclear aggregates also contained the scaffolding protein VP22a and the minor capsid proteins pUL17 and pUL25 (c.f. Table 1).

In our analysis, we used four different MAbs raised against VP5: LP12 and H1.4 that detect both, soluble VP5 and capsids, as well as 5C10 and 8F5 that recognize mature hexon epitopes on capsids whose formation requires ATP, proteolytic cleavage of the capsid scaffold and VP26 [5], [56], [62], [86]–[90]. However, only 5C10 labeled the entire aggregates whereas 8F5, LP12, and H1.4, and also the PAb anti-LC were restricted to their periphery, and after transfection with pHSV1(17^+^)blueLox-GFPVP26_Δaa**5**–7_ the 8F5 epitope could not be detected at all (c.f. Table 1). Chi & Wilson (2000) suggested that the tag on GFPVP26_Δaa**1**–7_ may interfere with 8F5 but not 5C10 binding, and we have also reported that 8F5 and 5C10 prefer untagged over HSV1(KOS)-GFPVP26_Δaa**1**–7_ capsids [5]. However, PAb anti-LC recognizes capsids irrespective of the tag, and LP12 even prefers HSV1(KOS)-GFPVP26_Δaa**1**–7_ over wild type capsids [5], whereas the nuclear aggregates described here exposed LC and LP12 epitopes only in their periphery. These immunological data are inconsistent with the notion that the nuclear aggregates are conglomerations of authentic progeny capsids. Furthermore, our correlative light and electron microscopy studies showed that many of the nuclear aggregates lack capsids. We therefore conclude that the aggregates are not some sort of capsid assemblons, but may represent an accumulation of dead-end products as also suggested by Lamberti & Weller (1998) and de Bruyn Kops et al. (1998) [69], [72]; nevertheless, they may abut areas enriched for capsids.

Therefore, in contrast to what we [8] and others have suggested, a recruitment of viral or host proteins to these nuclear aggregates containing VP5, VP22a, VP26, pUL17 and pUL25 does not indicate that they interact with authentic capsids. Our data rather suggest that these aggregates confiscate so much capsid proteins and possibly other proteins interacting with VP26 that proper capsid assembly and nuclear egress are no longer possible. In our experiments, even pUL36 had been sequestered by the nuclear aggregates in some few cells, although pUL36 usually does not associate with nuclear but only with cytoplasmic capsids [63]. Furthermore, Luxton et al. (2006) [91] reported that deleting UL36 in a PrV strain whose capsids have been tagged with GFPVP26 results in impaired nuclear egress whereas capsids of untagged PrV or HSV1 strains are still exported to the cytoplasm in the absence of pUL36 [63], [92]–[94]. Thus, two mutations with moderate phenotypes, namely a tag on VP26 and deletion of UL36, if combined might potentiate and result in a synthetic phenotype [45], [46], in this case impediment of nuclear egress. Such a crosstalk between different mutated proteins might be used to reveal subtle phenotypes which cannot be elucidated otherwise. Moreover, small chemical compounds fostering aggregation of VP26 might be developed into effective antiviral therapy that prevents HSV nuclear capsid egress and thus virion formation. On the other hand, for characterization of intracellular capsid trafficking, virion assembly and cell entry, we will base future tagging or disabling mutations in HSV1 proteins on HSV1(17^+^)-mRFPVP26_Δaa**1**–7_ that has a low propensity for nuclear aggregation, and therefore seems to contain a less invasive tag on VP26. Furthermore, its subcellular capsid distribution during the course of an infection resembles more that of untagged capsids when compared to the other tags on VP26.
